# Efficient Microwave-Assisted Synthesis of 5-Deazaflavine Derivatives 

**DOI:** 10.3390/molecules15107227

**Published:** 2010-10-20

**Authors:** Jorge Trilleras, Luis Gabriel López, Dency José Pacheco, Jairo Quiroga, Manuel Nogueras, José M. de la Torre, Justo Cobo

**Affiliations:** 1 Grupo de Investigación en Compuestos Heterocíclicos, Programa de Química, Facultad de Ciencias Básicas, Universidad del Atlántico, A.A.1890 Barranquilla, Colombia; 2 Grupo de Investigación de Compuestos Heterocíclicos, Departamento de Química, Universidad del Valle, A. A. 25360 Cali, Colombia; 3 Departamento de Química Inorgánica y Orgánica, Universidad de Jaén, 23071 Jaén, Spain

**Keywords:** pyrimidoquinolines, cyclocondensation, microwave, deazaflavines

## Abstract

A series of pyrimido[4,5-*b*]quinolines (5-deazaflavines), were synthesized by microwave assisted intramolecular cyclization. The *N*^4^-substituted-2,4-diamino-6-chloro-pyrimidine-5-carbaldehydes, were prepared by selective monoamination of 2-amino-4,6-dichloropyrimidine-5-carbaldehyde with aliphatic and aromatic amines.

## 1. Introduction

**Figure 1:**
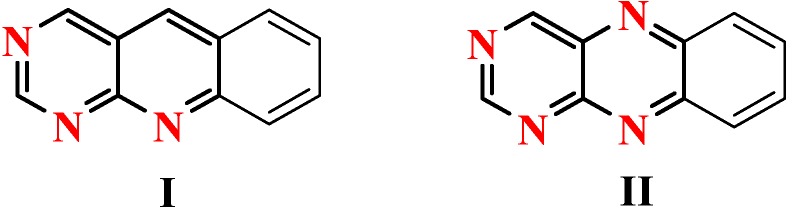
5-deazaflavine (pyrimido[4,5-*b*]quinoline) ring system **I** and pyrimido[4,5-*b*]quinoxaline ring system **II**.

The 5-deazaflavine (pyrimido[4,5-*b*]quinoline) ring system **I**is of great interest because of its structural similarity to the pyrimido[4,5-*b*]quinoxaline ring system of the naturally-occurring flavines (**II**, Figure 1), with N-5 being replaced by CH and thus keeping the redox properties of **I** quite similar to those of compounds **II**. Surprisingly, not much has been reported on the synthesis and properties of pyrimido[4,5-*b*]quinolines. Flavo-enzymes require flavine mononucleotide (FMN) or flavine adenine dinucleotide (FAD) as a coenzyme and catalyze oxidation-reduction reactions in biological systems [1,2]. Some heterocyclic compounds containing a quinoline moiety are of importance owing to their biological activities, especially antimalarial, antibacterial, analgesic and antitumor agents [3,4,5,6,7,8].

In addition to their pharmaceutical applications, they are attractive for physicochemical applications since they exhibit a high fluorescence in both solution and solid state under exposure to white light, which makes them appropriate candidates for the design of electroluminescent materials, like organic light-emitting diodes (OLEDs) [9,10,11,12,13,14,15].

Synthetic methods to prepare pyrimidoquinoline derivatives using the pyrimidine moiety [16,17,18,19,20] as starting material have been reported and involve a three-component reaction induced by microwave irradiation or by conventional heating. We have recently reported a straightforward *one-step* route to the 5-deazaflavin system *via* cyclocondensation of *N*^4^-aryl-2,4-diamino-6-chloropyrimidine-5-carbaldehydes used as unique starting materials [21].

## 2. Results and Discussion

**Figure 2:**
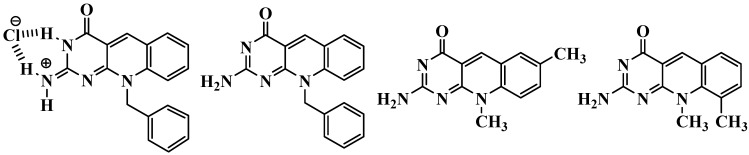
Pyrimido[4,5-*b*]quinoline derivatives synthesized.

Heterocyclic systems containing an aldehyde or ketone function in a position suitable for closing a six-membered ring permit the access to fused systems by treatment with acid *via* cyclodehydration that results in the formation of a double bond conjugated with the heteroaromatic ring [22]. Here, we describe three pyrimido[4,5-*b*]quinoline derivatives that were prepared in good yields using a straightforward synthesis (Figure 2).

**Scheme 1:**
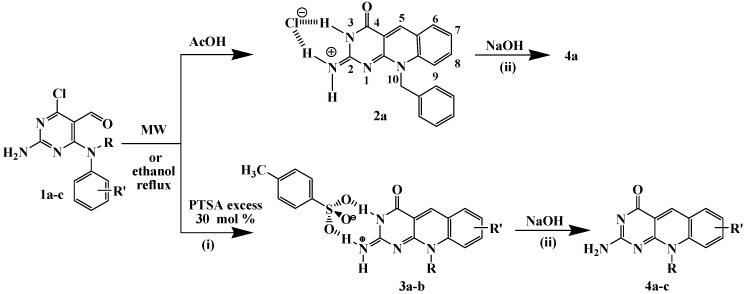
Synthesis of pyrimido[4,5-*b*]quinolines derivatives **2-4**.

In a first attempt *N*^4^–benzyl–*N*^4^–phenyl–2,4–diamino–6–chloropyrimidine–5–carbaldehyde (**1a**) and acetic acid were heated both under MW irradiation and by conventional heating. The reaction product corresponded to the deazaflavin analogue 10-benzyl-4-oxo-4,10-dihydropyrimido[4,5-*b*]quinolin-2(3*H*)-iminium chloride (**2a**), according to spectroscopic and MS analysis, that supposes the substitution of the chloro atom. Such a substitution appears to be entirely general in syntheses of this type, regardless of the nature of the acid employed [20,21]. Single crystal X-ray diffraction analysis of compound **2a** was used to corroborate the postulated structure [23]. Treatment of the salt **2a** with aqueous NaOH (20%) was carried out to give the neutral derivative **4a** in good yield (Scheme 1). 

**Scheme 2:**
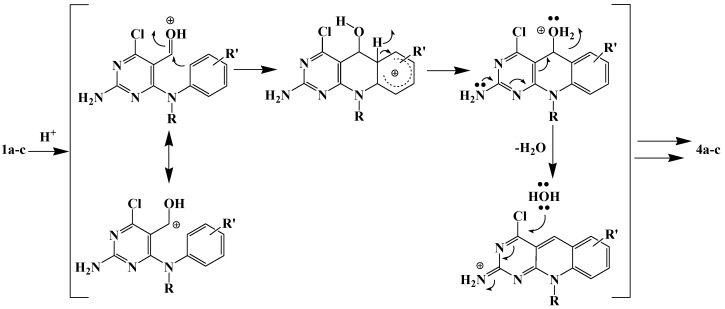
Acid-catalysed Bradsher-type cyclodehydration of diaryl aldehydes **1**.

Condensed aromatic systems are produced by cyclodehydration to give a double bond conjugated with the aromatic ring (Scheme 2). It seems likely that the acid is the most plausible source of the water component to nucleophilic aromatic substitution of the chloro atom by a hydroxyl group.

To avoid the substitution of the chloro atom in order to maintain the possibility of adding complexity and molecular diversity to the molecule, the same reaction was carried out using an excess of 4-toluenesulfonic acid (PTSA). Thus, **1a** (1.0 mmol) and an excess of PTSA monohydrate (1.3 mmol) were subjected to microwave irradiation (maximum power 300 W during 10 min at a controlled temperature of 573 K) using a focused microwave reactor (CEM Discover) or by conventional heating in refluxing ethanol. The reaction product was characterized from the spectroscopic data as the 1:1 salt 2-amino-10-benzylpyrimido[4,5-*b*]quinolin-4(10*H*)-one·PTSA (**3a**). The same type of salt **3b** was obtained using the 2,4-diamino-6-chloropyrimidine-5-carbaldehyde (**1b**). It is interesting to note that when the reaction was carried out by conventional heating of aldehydes **1** and acid (PTSA), reactions proceded rather similarly affording products **2a** and **3**. The only difference between those methods is that with microwave irradiation the reaction time is shorter than by conventional heating, 10 *vs.* 60 min, respectively (Table 1).

**Table 1:** The synthesized pyrimidoquinolines (deazaflavin analogues) *via* cyclocondensation of compounds **1** under microwave irradiation and reflux in ethanol.

Entry	Compound 1		Reaction conditions	Compound yield %					
	R	R’		MW (10 min)			Δ/Ethanol (60 min)		
				2a	3a-b	4a-c	2a	3a-b	4a-c
**a**	CH_2_C_6_H_5_	H	AcOH	80	-	-	85	-	-
			PTSA (1.3 mmol)	-	70	-	-	72	-
			(i) PTSA (1.3 mmol) (ii) NaOH	-	-	70	-	-	68
**b**	CH_3_	*p*-H_3_C	PTSA (1.3 mmol)	-	70	-	-	70	-
			(i) PTSA (1.3 mmol) (ii) NaOH	-	-	70	-	-	68
**c**	CH_3_	*o*-H_3_C	PTSA (0.2 mmol)	-	-	60	-	-	62

The ^1^H-NMR spectra of the salts **2a** and **3** are characterized by two singlets for the NH_2_ group protons as a result of the formation of the two cyclic N–H···Cl and N–H···O(PTSA) hydrogen bonds motifs, respectively. Treatment of the salts **2a** and **3** with aqueous NaOH (20%) was carried out to give the neutral derivatives **4a,b** in good yields. The derivative **4c** was obtained directly from the reaction between 4-(*N*-methyl-*N*-*o*-tolylamino)-2-amino-6-chloropyrimidine-5-carbaldehyde (**1c**) and a catalytic amount of PTSA (0.2 mmol), so a 1:1 ratio of acid is needed for the formation of corresponding salt.

## 3. Experimental

### 3.1. General

Melting points were determined in a Buchi Melting Point Apparatus and are reported uncorrected. The ^1^H- and ^13^C-NMR spectra were measured at RT on a Bruker Avance 400 spectrometer operating at 400 and 100 MHz, respectively, and using DMSO-*d_6_* as solvent and tetramethylsilane as internal standard. The mass-spectra were scanned on a Hewlett Packard HP Engine-5989 spectrometer (equipped with a direct inlet probe) which was operating at 70 eV. High Resolution Mass Spectra (HRMS) were recorded in a Waters Micromass AutoSpec NT spectrometer (STIUJA). The elemental analyses have been obtained using a LECO CHNS-900 and Thermo Finnigan FlashEA1112 CHNS-O (STIUJA) elemental analyzers.

*10-Benzyl-4-oxo-4,10-dihydropyrimido[4,5-b]quinolin-2(3H)-iminium chloride* (**2a**). *Microwave method*: A mixture of *N*^4^–benzyl–*N*^4^–phenyl–2,4–diamino–6–chloropyrimidine–5–carbaldehyde (**1a**, 1.0 mmol) and an excess of acetic acid (1.5 mL) were subjected to microwave irradiation (maximum power 300 W during 10 min at a controlled temperature of 573 K) using a focused microwave reactor (CEM Discover). The solid products were collected by filtration and washed with hot hexane to give yellow powder, yield 80%, m.p. > 300 ºC. ^1^H-NMR δ: 6.20 (s, 2H, CH_2_); 7.27–7.35 (m, 5H, CH phenyl); 7.68 (t, *J* = 7.5 Hz, 1H, H7); 7.93 (d, *J* = 8.8 Hz, 1H, H9); 8.01 (t, *J* = 8.3 Hz, 1H, H8); 8.39 (d, *J* = 8.0 Hz, 1H, H6), 8.54 (s, 1H, NH_2_), 9.19 (s, 1H, NH_2_), 9.45 (s, 1H, H5), 12.68 (s, 1H, NH). ^13^C –NMR δ: 48.9 (CH_2_); 116.2 (C4a); 118.3 (C9); 123.3 (C5a), 126.7 (C7), 127.1 CH*o* phenyl), 128.2 (CH*p* phenyl), 129.3 (CH*m* phenyl), 132.8 (C6); 135.3 (C*i* phenyl), 137.1 (C8), 139.9 (C9a); 144.9 (C5), 157.3 (C10a); 158.3 (C2); 160.1 (C4). IR (KBr) cm^-1 ^1712, 1654 (C=O *st*). MS (70 eV) *m/z* (%) = 302 (C_18_H_14_N_4_O, 99), 301 (78), 273 (13), 231 (30), 129 (14), 91 (100). Anal. Calcd for C_18_H_15_ClN_4_O: C, 63.81; H, 4.46; N, 16.54. Found: C, 63.34; H, 4.38; N, 16.61. *Conventional method*: A mixture of *N*^4^–benzyl–*N*^4^–phenyl–2,4–diamino–6–chloropyrimidine–5–carbaldehyde (**1a**, 1.0 mmol) and an excess of acetic acid (1.5 mL) were heated under reflux in ethanol during 60 min, then allowed to cool. The solid product was collected and washed with hot hexane to give the corresponding derivative. 

### 3.2. General procedure for the synthesis of pyrimido[4,5-b]quinolin-2(3H)-iminium-4-toluene-sulfonates **3**

*Microwave method*: A mixture of A mixture of *N*^4^-substituted-2,4-diamino-6-chloropyrimidine-5-carbaldehydes **1a,b** (1.0 mmol) and an excess of PTSA (1.3 mmol) were subjected to microwave irradiation (maximum power 300 W during 10 min at a controlled temperature of 573 K) using a focused microwave reactor (CEM Discover). The solid products were collected by filtration and washed with hot hexane to give the corresponding derivatives. *Conventional method*: A mixture of *N*^4^-substituted-2,4-diamino-6-chloropyrimidine-5-carbaldehydes **1a,b** (1.0 mmol) and an excess PTSA (1.3 mmol) were heated under reflux in ethanol during 60 min, then allowed to cool. The solid product was collected and washed with hot hexane to give the corresponding derivatives.

*10-Benzyl-4-oxo-4,10-dihydropyrimido[4,5-b]quinolin-2(3H)-iminium-4-toluenesulfonate* **(3a**). A yellow powder, yield 70%, m.p. > 300 ºC. ^1^H-NMR δ: 2.29 (s, 3H, H_3_C-PTSA), 6.21 (s, 2H, CH_2_), 7.12 (d, *J* = 8.0 Hz, 2H, H*m*’-PTSA), 7.27–7.33 (m, 5H, phenyl), 7.50 (d, *J* = 8.0 Hz, 2H, H*o*’-PTSA), 7.69 (t, *J* = 7.4 Hz, 1H, H7), 7.94 (d, *J* = 8.8 Hz, 1H, H6), 8.01 (t, *J* = 8.5 Hz, 1H, H8), 8.10 (s, 1H, NH_2_), 8.40 (d, *J* = 8.0 Hz, 1H, H9), 9.47 (s, 1H, H_5_), 9.16 (s, 1H, NH_2_), 12.39 (s, 1H, NH). ^13^C-NMR δ: 20.7 (CH_3_), 48.4 (CH_2_), 115.5 (C4a), 117.8 (C9), 122.8 (C5a), 125.4 (C*m*’-PTSA), 126.2 (C7), 126.6 (C*o*’-PTSA), 127.6 (C*p*), 128.0 (C*o*), 128.7 (C*m*), 132.3 (C6), 134.8 (C*i*), 136.7 (C8), 137.8 (C*p*’-PTSA), 139.4 (C9a), 144.3 (C5), 145.3 (C*i*’-PTSA), 156.8 (C10a), 157.4 (C4), 159.9 (C2). IR (KBr) cm^-1 ^1714 (C=O *st*), 1605 (C=C *st*), 1568 (NH *st*). MS (70 eV) *m/z* (%) = 302 (C_18_H_14_N_4_O, 30), 231 (10), 172 (PTSA, 10), 91 (100). Anal. Calcd for C_25_H_22_N_4_O_4_S: C, 63.28; H, 4.67; N, 11.81. Found: C, 63.18; H, 4.70; N, 11,93.

*7,10-Dimethyl-4-oxo-4,10-dihydropyrimido[4,5-b]quinolin-2(3H)-iminium-4-toluenesulfonate* (**3b**). A yellow powder, yield 70%, m.p. > 300 ºC. ^1^H-NMR δ: 2.28 (s, 3H, CH_3_ PTSA), 2.52 (s, 3H, 7-CH_3_), 4.25 (s, 3H, 10-CH_3_), 7.09 (d, *J* = 8.0 Hz, 2H, H*m*), 7.52 (d, *J* = 8.3 Hz, 2H, H*o*), 7.97 (d, *J* = 8.8 Hz, 1H, H8), 8.06 (d, *J* = 8.5 Hz, 1H, H9), 8.14 (s, 1H, H6), 8.36 (s, 1H, NH_2_), 9.24 (s, 1H, H5), 9.58 (s, 1H, NH_2_), 11.93 (s, 1H, NH). ^13^C-NMR δ: 20.1 (7-CH_3_), 20.6 (CH_3 _PTSA), 33.6 (CH_3_ N-10), 114.9 (C4a), 117.4 (C9), 122.7 (C5a), 125.5 (C*o*), 127.9 (C*m*), 130.9 (C6), 136.2 (C7), 137.5 (C*p*), 138.5 (C8), 138.9 (C9a), 143.5 (C5), 145.9 (C*i*), 155.8 (C10a),156.9 (C4), 159.6 (C2). IR (KBr) cm^-1^ 3407 (NH, *st*), 1709 (C=O, *st*), 1578 (NH, *st*). MS (70 eV) *m/z* (%) = 240 (C_13_H_12_N_4_O, 64), 212 (45), 172 (PTSA, 30), 91 (100). Anal. Calcd for C_20_H_20_N_4_SO_4_: C, 66.09; H, 5.27; N, 15.42. Found: C, 66.01; H, 5.20; N, 14.45.

Treatment of the salts **2a** and **3** with aqueous NaOH (20%) was carried out to afford the neutral derivatives **4** in good yields.

*2-Amino-10-benzylpyrimido[4,5-b]quinolin-4(10H)-one* (**4a**). A yellow powder, yield 70%, m.p. > 300 ºC. ^1^H-NMR δ: 6.06 (s, 2H, CH_2_), 6.97 (s, 2H, NH_2_), 7.23–7.25 (m, 3H, H*o,* H*p*), 7.30 (t, *J* = 7.5 Hz, 2H, H*m*), 7.45 (t, *J* = 7.78 Hz, 1H. H7), 7.70 (d, *J* = 8.78 Hz, 1H, H6), 7.77 (t, *J* = 7.28 Hz, 1H, H8), 8.14 (d, *J* = 7.78 Hz, 1H, H9), 8.90 (s, 1H, H5). ^13^C-NMR δ: 46.6 (CH_2_), 116.1 (C6), 116.6 (C4a), 121.8 (C5a), 123.7 (C7), 126.2 (C*o*), 126.8 (C*p*), 128.2 (C*m*), 131.1 (C9), 133.6 (C8), 135.4 (C*i*), 138.8 (C9a), 140.0 (C5), 158.2 (C10a), 168.3 (C2), 168.8 (C4). IR (KBr) cm^-1 ^3391 (NH, *st*), 1636 (C=O *st*). MS (70 eV) *m/z* (%) = 302 (M^+^, 45), 91 (100). Anal. Calcd for C_18_H_14_N_4_O: C, 71.51; H, 4.67; N, 18.53. Found: C, 71.47; H, 4.62; N, 18.55.

*2-Amino-7,10-dimethylpyrimido[4,5-b]quinolin-4(10H)-one* **(4b).** A yellow powder, yield 70%, m.p. > 300 ºC. ^1^H-NMR δ: 2.46 (s, 3H, 7-CH_3_), 4.07 (s, 3H, 10-CH_3_), 6.84 (s, 2H, NH_2_), 7.73 (d, *J* = 7.3 Hz, 1H, H8), 7.80 (d, *J* = 8.5 Hz, 1H, H9), 7.91 (s, 1H, H6), 8.75 (s, 1H, H5). ^13^C-NMR δ: 20.3 (7-CH_3_), 32.4 (10-CH_3_), 116.6 (C6), 122.3 (C5a), 130.7 (C9), 134.4 (C7), 136.5 (C8), 138.6 (C9a), 140.6 (C5), 157.7 (C10a). IR (KBr) cm^-1^ 3446 (NH, *st*), 1651 (C=O, *st*). MS (70 eV) *m/z* (%) = 240 (M^+^, 84), 212 (62), 77 (45), 28 (100). Anal. Calcd for C_13_H_12_N_4_O: C, 64.99; H, 5.03; N, 23.32. Found: C, 65.03; H, 5.10; N, 23.28.

*2-Amino-9,10-dimethylpyrimido[4,5-b]quinolin-4(10H)-one* (**4c**). A yellow powder, yield 60%, m.p. > 300 ºC. ^1^H-NMR δ: 2.48 (s, 3H, 9-CH_3_ ), 4.18 (s, 3H, 10-CH_3_), 6.79 (s, 2H, NH_2_), 7.37 (t, *J* = 7.53 Hz, 1H, H7), 7.67 (d, *J* = 7.03 Hz, 1H, H8), 7.91 (d, *J* = 7.53 Hz, 1H, H6), 8.72 (s, 1H, H5). ^13^C-NMR δ: 23.1 (9-CH_3_), 38.0 (10-CH_3_), 115.9 (C4a), 123.1 (C5a), 123.5 (C7), 126.1 (C9), 129.1 (C8), 137.7 (C6), 139.9 (C5), 140.6 (C9a), 159.8 (C10a), 166.9 (C2), 168.3 (C4). IR (KBr) cm^-1^ 3419 (NH, *st*), 1644 (C=O, *st*). MS (70 eV) *m/z* (%) = 240 (M^+^, 15), 212 (7), 105 (64), 77 (100). Anal. Calcd for C_13_H_12_N_4_O: C, 64.99; H, 5.03; N, 23.32. Found: C, 64.93; H, 4.98; N, 23.35.

## 4. Conclusions

In this work we are describing the synthesis of pyrimidoquinolines (deazaflavin analogues), *via* a simple, efficient, and versatile *one-step* method assisted by microwave irradiation. The reaction offers a strategy for the preparation of quinolines from *N*^4^-substituted-2,4-diamino-6-chloropyrimidine-5-carbaldehydes. Compared with other methods, this one has the advantages of high yields, mild reaction conditions, easy work-up, inexpensive reagents, and an environmentally friendly procedure. The chemical (fluorescence) and biological (antifungal and antitumor) properties of the new compounds obtained in these experiments are under investigation. 
